# Enhanced l-Malic Acid Production by *Aspergillus oryzae* DSM 1863 Using Repeated-Batch Cultivation

**DOI:** 10.3389/fbioe.2021.760500

**Published:** 2022-01-10

**Authors:** Vanessa Schmitt, Laura Derenbach, Katrin Ochsenreither

**Affiliations:** Institute of Process Engineering in Life Sciences 2: Technical Biology, Karlsruhe Institute of Technology (KIT), Karlsruhe, Germany

**Keywords:** organic acids, malic acid, *Aspergillus oryzae*, fungi, batch, repeated-batch, fed-batch, productivity

## Abstract

l-Malic acid is a C4-dicarboxylic acid and a potential key building block for a bio-based economy. At present, malic acid is synthesized petrochemically and its major market is the food and beverages industry. In future, malic acid might also serve as a building block for biopolymers or even replace the commodity chemical maleic anhydride. For a sustainable production of l-malic acid from renewable resources, the microbial synthesis by the mold *Aspergillus oryzae* is one possible route*.* As CO_2_ fixation is involved in the biosynthesis, high yields are possible, and at the same time greenhouse gases can be reduced. In order to enhance the production potential of the wild-type strain *Aspergillus oryzae* DSM 1863, process characteristics were studied in shake flasks, comparing batch, fed-batch, and repeated-batch cultivations. In the batch process, a prolonged cultivation time led to malic acid consumption. Keeping carbon source concentration on a high level by pulsed feeding could prolong cell viability and cultivation time, however, did not result in significant higher product levels. In contrast, continuous malic acid production could be achieved over six exchange cycles and a total fermentation time of 19 days in repeated-batch cultivations. Up to 178 g/L l-malic acid was produced. The maximum productivity (0.90 ± 0.05 g/L/h) achieved in the repeated-batch cultivation had more than doubled than that achieved in the batch process and also the average productivity (0.42 ± 0.03 g/L/h for five exchange cycles and 16 days) was increased considerably. Further repeated-batch experiments confirmed a positive effect of regular calcium carbonate additions on pH stability and malic acid synthesis. Besides calcium carbonate, nitrogen supplementation proved to be essential for the prolonged malic acid production in repeated-batch. As prolonged malic acid production was only observed in cultivations with product removal, product inhibition seems to be the major limiting factor for malic acid production by the wild-type strain. This study provides a systematic comparison of different process strategies under consideration of major influencing factors and thereby delivers important insights into natural l-malic acid production.

## Introduction

Over the past decades, concerns about climate change and oil depletion led to a growing interest in the idea of a bio-based economy, in which fuels, chemicals, and pharmaceuticals are produced from renewable resources *via* eco-friendly synthesis routes. In 2004, malic acid was identified as one potential key building block for the bio-economy (Werpy et al., 2004). As a dicarboxylic acid with an additional hydroxy group, malic acid has several established applications in the food and beverages industry, as a buffer and chelating agent, and in the pharmaceutical industry. Furthermore, it might be applied as a building block for homo- and heteropolymers, for biomedical applications, such as drug carriers, and as a component in low transition temperature mixtures for eco-friendly extraction purposes. Moreover, it has the potential to replace the established platform chemical maleic anhydride (Koevilein et al., 2020).

Malic acid is mainly produced chemically as a racemic mixture from fossil resources with an estimated global market volume of 80,000–100,000 ton per year (Koevilein et al., 2020). In contrast, enantiopure l-malic acid (physiological form) can be exclusively produced enzymatically or by microbial synthesis. Advantageous in this case is the classification as “nature identical” (NATRUE lable, https://www.natrue.org/), which results in a higher sales value and is needed for pharmaceutical applications. The polymer of l-malic acid is also adsorbed differently than a polymer of the racemate when used as a carrier material for pharmaceuticals (Lee et al., 2011). In addition, homo- and heteropolymers derived from l-, d-, or dl-malic acid might have different characteristics since the racemate and both enantiomers vary in melting and boiling point (Fiume, 2001).

A sustainable route for the l-malic acid production is the microbial fermentation. Although l-malic acid is part of the TCA cycle, its accumulation and secretion into culture broth was only observed under certain stress conditions in a few filamentous fungi and yeasts, such as *Aspergillus* and *Ustilago* species (Koevilein et al., 2020). These fungi accumulate l-malic acid *via* a reductive branch of the TCA cycle located in the cytosol, under nitrogen limitation and simultaneous excess of the carbon source. The biosynthesis is ATP neutral and includes a CO_2_ fixation step, resulting in a theoretical yield of 2 mol l-malic acid per mol glucose (Zelle et al., 2008).

Among the genus *Aspergillus*, *A. oryzae* is a very promising natural l-malic acid producer for industrial scale fermentations, due to the combination of strain safety, robustness, wide substrate range, and a high production capacity (Koevilein et al., 2020). However, l-malic acid production from renewable resources is still missing the economic competitiveness to substitute petrochemical synthesis routes. The key aspects for increasing productivities and yields and therefore economic viability of biotechnological processes are optimization of media and process conditions as well as genetical engineering.

In terms of media optimization, the nitrogen source and the C:N ratio have been identified as important aspects for the l-malic acid production with *A. oryzae.* According to Ding et al. much higher malic acid titers can be achieved by using complex nitrogen sources such as tryptone instead of ammonium sulfate. Furthermore, malic acid production can be increased by optimization and control of the C:N ratio (Ochsenreither et al., 2014; Liu et al., 2017; Ding et al., 2018; Ji et al., 2021). Another study proved the importance of calcium carbonate supply for pH stability and malic acid production (Geyer et al., 2018).

Concerning genetical engineering, l-malic acid production was improved considerably by strengthening the malic acid export due to the overexpression of a C4-dicarboxylate transporter gene from *A. oryzae* and the l-malate permease gene from *Schizosaccharomyces pombe* (Brown et al., 2013; Liu et al., 2017; Liu et al., 2018). The genetical modifications introduced by Brown et al. (2013) resulted in the highest reported yields, so far. In 2 L bioreactor batch cultivations on 100 g glucose and 6 g peptone, a yield of 1.00 g/g or 1.38 mol/mol was reached after 164 h, corresponding to 69% of the theoretical maximum. The best results for malic acid production using ammonium sulfate as a nitrogen source were achieved by Ding et al. in 7.5 L fed-batch with a mutant strain of *A. oryzae*. On 176 g/L glucose and 5.5 g/L (NH_4_)_2_SO_4,_ a titer of 95.2 g/L l-malic acid was achieved, corresponding to productivity of 0.57 g/L/h and a yield of 0.54 g/g or 0.73 mol/mol.

It is noticeable that for the l-malic acid production with natural producers, mostly batch processes and only occasionally fed-batch processes are applied. To our knowledge, there is only one study, in which batch and fed-batch cultivations of *A. oryzae* are directly compared regarding the malic acid synthesis. Liu et al. (2017) tested an automated fed-batch process for a genetically modified *A. oryzae* high-performance strain and compared it to batch fermentation. In this 3 L fed-batch cultivation, the l-malic acid concentration reached a maximum of 165 g/L after 120 h, resulting in a productivity of 1.38 g/L/h. This is the highest malic acid titer achieved with *A. oryzae*. In contrast, only 73 g/L l-malic acid was produced in the batch process until glucose depletion after 60 h. A fed-batch process was also applied for the l-malic acid production with *U. trichophora* using large quantities of glycerol (Zambanini et al., 2016), probably due to the high viscosity of the substrate.

In contrast, data for repeated-batch processes are only available for fungal production of other organic acids. Advantages, such as prolonged product synthesis and increased productivities, were observed in fungal repeated-batch cultivations for the production of organic acids, such as kojic acid (Wan et al., 2005), fumaric acid (Petruccioli et al., 1996), itaconic acid (Park et al., 1994; Hosseinpour Tehrani et al., 2019), citric acid (Roukas, 1991; Sakurai and Imai, 1992; Yu et al., 2018), lactic acid (Yang et al., 1995; Yin et al., 1998), and isocitric acid (Morgunov et al., 2019). As a reason for the improved acid synthesis, a reduction of product inhibition is assumed. Accordingly, repeated-batch cultivation should also be beneficial for l-malic acid production with *A. oryzae*.

This study aims to compare systematically different cultivation strategies for the l-malic acid synthesis by the wild-type *A. oryzae* DSM 1863. For this purpose, process characteristics of batch, fed-batch, and repeated-batch cultivations were determined in parallel shake flask experiments. Thereby, the study illuminates the question whether the high productivities, observed in genetically modified organisms (GMOs) with overexpressed transporter genes, can also be achieved with a wild-type strain by changing the process mode. Hence, different feeding regimes for fed-batch and repeated-batch cultivations were tested, and the overall produced malic acid amount, productivities, and yields were compared to batch fermentations. Additionally, the study provides new insights into the role of nitrogen and calcium carbonate for prolonged l-malic acid production processes.

## Materials and Methods

### Chemicals

All chemicals were either purchased from Sigma-Aldrich (Munich, Germany) or Roth (Karlsruhe, Germany).

### General Cultivation Aspects: Fungi, Media, and Cultivation Conditions

The fungal strain *A. oryzae* DSM 1863 was obtained from the DSMZ strain collection (Deutsche Sammlung von Mikroorganismen und Zellkulturen GmbH, Braunschweig, Germany). The strain was treated as described by Dörsam et al. (Dörsam et al., 2016), and the generated conidia aliquots in glycerin were stored at −80°C until further usage.

The l-malic acid production was performed in a two-step process, including a pre-culture and a main culture medium. The pre-culture medium consists of 40 g/L glucose monohydrate, 4 g/L (NH_4_)_2_SO_4_, 0.75 g/L KH_2_PO_4_, 0.98 g/L K_2_HPO_4_ 3H_2_O, 0.1 g/L MgSO_4_ 7H_2_O, 0.1 g/L CaCl_2_ 2H_2_O, 0.005 g/L NaCl, and 0.005 g/L FeSO_4_ 7H_2_O. The main culture medium comprises 120 g/L glucose monohydrate, 1.2 g/L (NH_4_)_2_SO_4_, 0.1 g/L KH_2_PO_4_, 0.17 g/L K_2_HPO_4_ 3H_2_O, 0.1 g/L MgSO_4_ 7H_2_O, 0.1 g/L CaCl_2_ 2H_2_O, 0.005 g/L NaCl, and 0.060 g/L FeSO_4_ 7H_2_O. The media were sterilized by autoclaving. For pH regulation, sterile 90 g/L CaCO_3_ was added to the autoclaved main culture medium.

The fermentation process for l-malic acid production with *A. oryzae* was carried out in 500-ml baffled Erlenmeyer shake flasks with a working volume of 100 ml. The pre-culture was inoculated with 2 × 10^7^ conidia and incubated at 100 rpm and 30°C for 24 h in a rotary shaker with a shaking diameter of 25 mm. Next, the pre-culture medium was removed; the fungal pellets were washed twice and resuspended in 100 ml water before being transferred to the main culture medium. The shake flasks were inoculated with 10% (v/v) washed pre-culture and incubated at 120 rpm and 32 or 35°C (Table 1).

**TABLE 1:** Overview of the experimental conditions.

Experiment no. and general conditions	Cultivation name/process mode	Addition of CaCO_3_ ^a^	Feed/exchange medium
No. 1 Feeding and exchange rate^b^: every 3 days; temperature: 35°C	Batch A	—	—
	FB A	—	Glucose
	FB B	—	Glucose and N-source
	FB C	—	Complete medium**^c^**
	RB A	6 g after every exchange	Complete medium**^c^**
	RB B	3 g after every exchange	Complete medium**^c^**
No. 2 Feeding and exchange rate^b^: every 2 days; temperature: 32°C	Batch B	—	—
	RB C	Regular additions of 2.5 g as needed^d^	Complete medium**^c^**
	RB D	t = 336 h: 9 g and t > 336 h: Regular additions of 2.5 g as needed^d^	Complete medium**^c^**
	RB E	Regular additions of 2.5 g as needed^d^	Complete medium**^c^** and without N-source

a Starting concentration of calcium carbonate of all cultivations was 90 g/L.

b Feeding and exchange rates after the initial growth phase of 4 days.

c The “complete medium” comprises all components of the batch medium as listed in the Materials and Methods section, except for sodium chloride.

d “As needed” means that calcium carbonate was only added in case the medium has cleared up.

### l-Malic Acid Production

In the first experiment, five shake flask cultivations were carried out in parallel: one batch (Batch A), two repeated-batch (RB A/B), and three fed-batch fermentations (FB A/B/C). In the repeated-batch approach, the addition of different amounts of calcium carbonate with every media exchange was compared (6 g CaCO_3_ in RB A vs. 3 g in RB B). In the fed-batch process, three different feed compositions were tested. Feeding or medium replacement in the first parallel cultivation experiment was performed every third day. For further evaluation of the repeated-batch process, another set of experiments was conducted, including a batch process (Batch B) as a control and three different repeated-batch scenarios (RB C/D/E) with a 2-day medium exchange regime. This second set of fermentations (RB C/D/E) was run at 32°C. Besides the more frequent medium replacements, the role of calcium carbonate and ammonium was evaluated by omitting the addition of one of the two chemicals during the cultivation in RB D and E, respectively.

All experiments (Table 1) were performed as triplicate with three individual shake flasks for each tested condition. The cultivations were run for a minimum of at least 2 weeks (Batch A) to a maximum of 23 days (RB C). For sampling, 5 ml from the fermentation broth was transferred to a sampling vessel, excluding fungal pellets. After pH measurement, the samples were stored at −20°C until HPLC analysis. After initial sampling prior incubation, no samples were taken in the first 72 h of the fermentation. Afterward, regular sampling was performed, depending on the needs of the cultivation process. In batch fermentations, samples were taken every 24–48 h. In the fed-batch and repeated-batch cultivations, samples were also taken prior and directly after each feeding or medium replacement.

In the fed-batch approach, feed volume was chosen to be 20 ml to compensate the volume loss due to sampling. The glucose concentration of all feed solutions was 250 g/L, except for the first feed with only 200 g/L, assuming lower glucose consumption during the growth phase at the beginning of the cultivation. While in FB A, the feed solely includes glucose, all main culture medium components, except for NaCl, were incorporated in FB C. The feeds of FB C comprised 6 g/L (NH_4_)_2_SO_4_, 0.5 g/L KH_2_PO_4_, 0.75 g/L K_2_HPO_4_·3H_2_O, 0.5 g/LMgSO_4_ 7H_2_O, 0.5 g/L CaCl_2_ 2H_2_O, 0.3 mg/L FeSO_4_ 7H_2_O, and glucose with the aforementioned variable concentration. FB B combines a carbon and nitrogen feed, adding 6 g/L (NH_4_)_2_SO_4_ to the glucose solution.

In repeated-batch cultivations, the medium was removed by decanting, and shake flasks were refilled with the 100 ml fresh medium. In RB A, B, C, and D, the fresh medium had the same composition as the initial main culture medium, whereas in RB E ammonium sulfate was omitted from the exchange medium. The medium of the first batch included in all cultivations contained 9 g CaCO_3_. Additional calcium carbonate was regularly added in all repeated-batch cultivations, except for RB D, to supplement the loss of calcium carbonate caused by the exchange procedure. While calcium carbonate was added after each medium replacement in RB A (6 g) and RB B (3 g), in RB C and E, 2.5 g CaCO_3_ was only added to the fresh medium in case the medium has become quite clear. In RB E, the regular calcium carbonate additions were omitted, and 9 g CaCO_3_ was added twice; at the beginning of the experiment and after the l-malic acid production had virtually stopped. Afterward, calcium carbonate (2.5 g) was added only in case the medium has become quite clear.

### l-Malic Acid and Glucose Analytics

The quantification of l-malic acid and glucose was carried out with a high-performance liquid chromatography (HPLC) using an Agilent 1100 Series LC system (Agilent, Germany).

The glucose concentration was determined in the supernatant of the fermentation broth samples after centrifugation for 5 min at 20,000 × g in a benchtop device. HPLC was equipped with a Rezex™ ROA-Organic Acid H+ (8%) LC Column (300 x 7.8, Phenomenex, Aschaffenburg, Germany) and a pre-connected Rezex™ ROA-Organic Acid H+ Guard Column (50 × 7.8 mm, Phenomenex). Glucose was detected *via* a refractive index detector (Agilent 1200 Series, Agilent, Germany). The analysis was performed under isocratic conditions at a column temperature of 50°C for 20 min with 5 mM H_2_SO_4_ as the mobile phase and at a constant flow rate of 0.5 ml/min. The injection volume was 10 μl.

For malic acid quantification, 1 ml culture broth was mixed with 1 ml 3 M H_2_SO_4_ and 3 ml water and heated to 80°C for 20 min to resolve the precipitated calcium malate. After centrifugation of the mixture for 5 min at 20,000 × g, the supernatant was analyzed *via* HPLC. The device was equipped with a Synergi™ 4 μm Fusion-RP 80Å LC Column (150 × 4.6 mm, Phenomenex, Aschaffenburg, Germany). The analysis was performed at a column temperature of 30°C and a flow rate of 1 ml/min. The following gradient was applied using 100% methanol (eluant A) and 20 mM KH_2_PO_4_ with a pH of 2.5 (eluant B): 0–0.5 min 100% eluant B, 0.5–10 min increase of eluant A from 0–10%, 10–12 min decrease of eluant A from 10% back to 0%, and 12–14 min again 100% eluant B. Malic acid was detected *via* UV at a wavelength of 220 nm. The injection volume was 10 μl.

## Results

Previously, we reported that *A. oryzae* DSM 1863 is a promising natural l-malic acid producer with a wide substrate range (Ochsenreither et al., 2014; Dörsam et al., 2017; Kövilein et al., 2021). For further process understanding and optimization of malic acid production, we compared the three fermentation strategies, batch, fed-batch, and repeated-batch, in parallel shake flask cultivations. A variety of different experimental setups was tested in triplicate.

### Characterization of l-Malic Acid Production in Batch Cultivations

**FIGURE 1:**
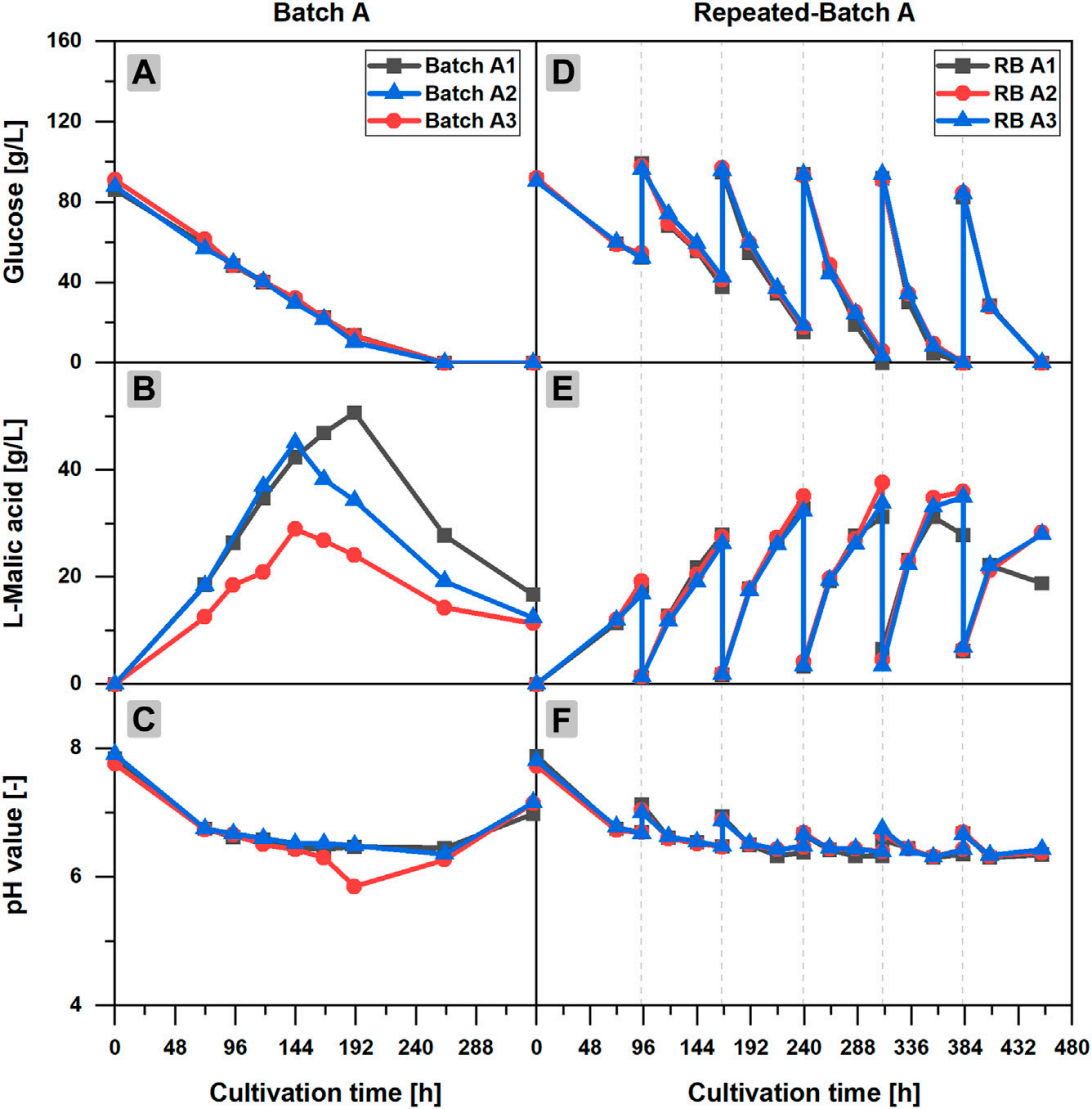
l-Malic acid production in batch and repeated-batch cultivations of *Aspergillus oryzae* DSM 1863. Course of the glucose concentration **(A,D)**, the l-malic acid concentration **(B,E)**, the pH value **(C,F)** of Batch A **(A–C),** and repeated-batch A **(D–F)** over the cultivation time. The triplicate data A1**–**3 of the batch and repeated-batch cultivations are distinguishable by color and symbol. The gray vertical, dotted lines indicate the times of medium exchange for the repeated-batch cultivation.

The l-malic acid production in batch cultivations can be divided into three phases (Batch A1-3 in Figures 1A–C): the fungal growth phase, the major production phase, and a final malic acid reduction phase. In the first 72 h of cultivation, when fungal pellets grow, glucose and ammonium are consumed and malic acid synthesis starts; a steep decrease of the pH value from 7.84 ± 0.08 to 6.75 ± 0.01 occurred (Figure 1C). From further experiments with daily sampling between 0 and 72 h, it is known that the pH drop has happened already in the first 48 h until ammonium is depleted (data not shown). After the initial growth phase, a slow but continuous decline of the pH value over time could be observed, when the malic acid production accelerated. The triplicate showed significant deviations with regard to the maximum malic acid titers and the time points, at which the maximum product concentration was measured (Figure 1B). However, in each individual batch cultivation, malic acid concentration clearly peaked, followed by a steep decline afterward, presumably due to product metabolization. For the glucose concentration, the triplicate data were much more consistent. In all cultivations, glucose was fully consumed after 263 h of incubation (Figure 1A). Starting with an initial concentration of 88.3 ± 2.1 g/L, glucose was consumed continuously with an average glucose consumption rate of 0.40 ± 0.05 g/L/h in the first 191 h of cultivation. Accordingly, the estimated time of glucose depletion was 222 h. The highest l-malic acid titer was measured in Batch A1 with 50.7 g/L after 191 h. But the maximum might have been even higher, as no sample was taken at the calculated glucose depletion time. In contrast, malic acid production in Batch A2 and Batch A3 stopped before glucose was totally consumed, reaching maximum product concentrations of 45.1 g/L and 29.0 g/L, respectively, after 144 h and leaving about 30 g/L of residual glucose. Despite different time points for the maxima, a similar increase of the malic acid concentration could be observed in Batch A1 and Batch A2 until 144 h of cultivation. In contrast, the malic acid synthesis in Batch A3 was slower, right from the start of the cultivation, although the glucose consumption was comparable in all batches. Batch A3 also showed deviations with regard to the pH value, in particular the pH drops from 6.30 after 166 h to a minimum of 5.85 after 191 h (Figures 1C). Nevertheless, the course of the pH value was similar for all batches. The slow decline of the pH value during the major malic acid production phase continued after the malic acid concentration peaked, reaching a minimum of 6.36 ± 0.09 after 263 h, disregarding the pH drop of Batch A3 at 191 h. The end of the fermentation was characterized by a steep increase of the pH to 7.10 ± 0.10 after 336 h and an increasing number of fragmented fungal pellets.

For a later comparison to the other process mode experiments, yields, productivities, and glucose consumption rates were calculated based on glucose and l-malic acid concentrations after 144 h (Table 2).

**TABLE 2:** Comparison of process modes for l-malic acid production with *A. oryzae* DSM 1863 in 100-ml shake flasks.

Process mode^a^	Time^b^ (h)	CaCO_3_ addition^c^ (g)	MA productivity^d^ (g)	SD (g)	Glucose consumption rate^d^ (g)	SD (g)	Yield (g/g)	SD (g)	pH min (-)	SD (-)
Batch A	144	—	3.88	0.70	5.74	0.17	0.68	0.13	6.36^e^	0.09
RB A	453	6	17.80	0.42	44.05	0.54	0.40	0.01	6.31	0.01
RB B	453	3	15.02	0.81	43.24	0.73	0.35	0.02	6.09	0.01
FB C	453	—	4.67	0.29	41.90	0.33	0.11	0.01	5.19	0.05

a Best performing experiments for the individual process modes batch, repeated-batch (RB), and fed-batch (FB) were selected for comparison of the process modes based on specific process characteristics.

b For Batch A, the cultivation time until the maximum product titer is given (total cultivation time 333 h). For RB A/B and FB C, the overall cultivation time is given.

c Regular additions of calcium carbonate during cultivation. Starting concentration of calcium carbonate of all cultivations was 90 g/L and is not included.

d Overall l-malic acid/glucose amount produced/consumed as sum of all repeated-batch or fed-batch cycles. For Batch A, the amount of l-malic acid/glucose produced/consumed until 144 h is given.

e Average pH value minimum (after 263 h), disregarding the pH drop of Batch A3 at 191 h.

### Longer Productive Phase in Repeated-Batch Cultivations

Two repeated-batch cultivations were performed with regular additions of either 6 g (RB A) or 3 g (RB B) CaCO_3_ to the fermentation broth after each medium replacement. In both fermentations, l-malic acid was continuously synthesized in considerable amounts throughout the 453 h (19 days) of cultivation. However, with 178.0 ± 4.2 g/L, the total l-malic acid production was the highest for the cultivation with regular additions of 6 g CaCO_3_ (RB A). Accordingly, the overall glucose consumption in cultivations with the larger calcium carbonate dosage also exceeded (Table 2) the substrate consumption in the fermentations with only 3 g CaCO_3_ (RB B). Regarding pH, the reduced calcium carbonate supplementation resulted in lower values, especially in the second half of the cultivation, and with 6.09 in a lower pH minimum (Table 2). However, increasing the calcium carbonate additions also had a drawback: More solid particles result in a higher viscosity of the culture broth, thus complicating medium exchange. Despite these differences, the similarities prevailed, which is why only RB A is described in detail in the following sections.

Time courses of pH value, and l-malic acid and glucose concentration of RB A are characterized by the typical zigzag pattern of repeated-batch cultivations (Figures 1D–F). A closer look at the product curve reveals an acceleration of the malic acid production after each medium replacement from repeated-batch phase one to five. Due to the medium exchange procedure *via* decantation, the medium was not removed entirely, and the initial l-malic acid concentration of the repeated batches was increased from 1.6 ± 0.1 g/L after the first medium exchange to 7.2 ± 0.3 g/L after the last medium replacement. However, malic acid production clearly exceeded the rise of the initial concentrations. The l-malic acid titers at the end of each repeated-batch phase increased from 21.2 ± 1.2 g/L in the first phase to a maximum of 36.8 ± 1.4 g/L in phase three.

The high consistency of the triplicate data is remarkable. Only after glucose became a limiting factor in phase four, malic acid titers started to differ considerably. Although the initial glucose concentrations of each repeated-batch phase remained almost constant, a decline of the residual glucose at the end of each batch phase was observed over time due to an acceleration of the glucose consumption. Thus, glucose was completely consumed for the first time in phase three. Varying times for glucose depletion led to different malic acid maxima. While the maximum titer of repeated-batch A1 was measured alongside with glucose depletion at the end of the third phase, small amounts of residual glucose could still be detected in the other two cultivations. In phase four, glucose was completely consumed in each flask of the triplicate set. Accordingly, repeated-batch A2 reached the maximum titer in the fourth phase, whereas in repeated-batch A3 malic acid concentration was marginally higher in phase five instead of phase four. In the last repeated-batch phase, malic acid concentration was considerably reduced in all three fermentations.

With regard to the pH value, the triplicate data were highly consistent throughout the fermentation. The pH increased with every medium exchange and calcium carbonate addition. Within the individual phases of the repeated-batch cultivation, the course of the pH value was characterized by a pH drop in the first 24 h after medium replacement, followed by a slower decline of the pH value afterward. With 6.31 ± 0.01, the pH minimum of the repeated-batch cultivation was similar to the pH minimum of the batch process. Regarding the biomass, a considerable increase of the pellet sizes was observed over time, indicating continuous cell growth in the repeated-batch cultivations, fueled by the steady nitrogen supply.

### Comparison of Fed-Batch Cultivations With Different Feed Compositions

Three different feed compositions were tested in fed-batch cultivations, but l-malic acid production could not be enhanced compared to the batch fermentation. However, a better process understanding can be obtained by looking at the results of the individual feeding experiments.

**FIGURE 2:**
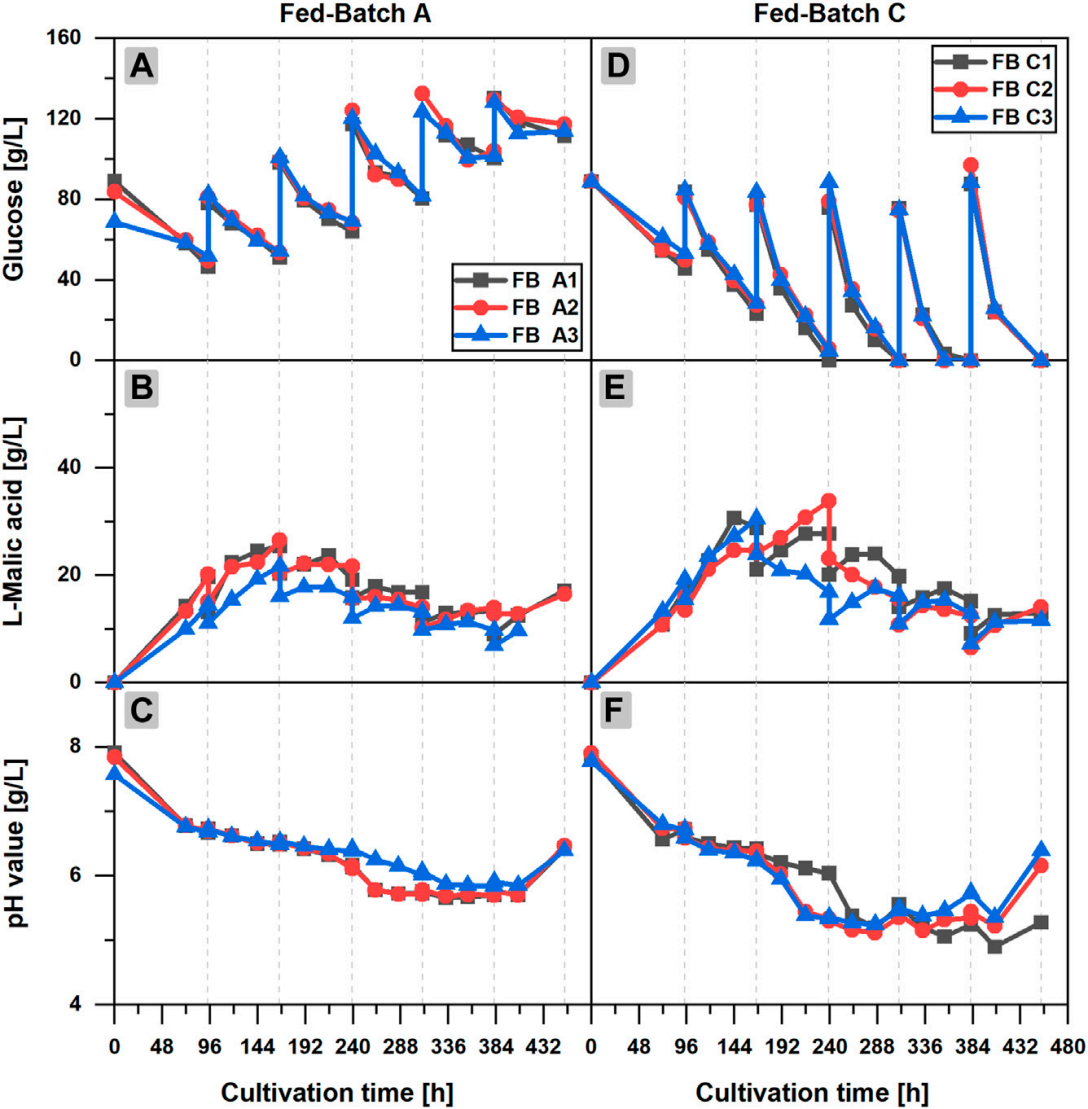
l-Malic acid production in fed-batch cultivations of *Aspergillus oryzae* DSM 1863. Comparison of fed-batch A with a glucose feed **(A–C)** and fed-batch C with a complete medium feed **(D–F)**.Course of the glucose concentration **(A,D)**, the l-malic acid concentration **(B,E)** and the pH value **(C,F)** over the cultivation time. The triplicate data A1**–**3 and C 1**–**3 of the fed-batch cultivations are distinguishable by color and symbol. The gray vertical, dotted lines indicate the feeding times.

In fed-batch scenario A (FB A) (Figures 2A–C), glucose depletion could be avoided by regular additions of concentrated glucose solution to the cultivation broth. More than 40 g/L glucose was available at any time of the cultivation in each flask of the triplicate set, but the l-malic acid concentration already reached a maximum of 26.5 g/L (FB A2) after 166 h, (3 days) after the first feeding. A longer cultivation with additional feedings did not increase the malic acid titer, although glucose was continuously consumed until the end of the fermentation. On the contrary, malic acid concentration was reduced in the further course of the fermentation, due to the dilution effect of the feedings and a net zero production of malic acid in several fed-batch phases. In phase 3–6, malic acid concentration increased slightly for 24 or 48 h after each feeding, staying constant or even decreasing afterward. The only exception was a slight increase of the malic acid concentration at the very end of the cultivation, probably caused by cell fragmentation and liberation of intracellular malic acid.

Regarding the glucose concentration, an accumulation of the substrate could be observed over time. Obviously, the feeding exceeded the glucose consumption. The pH value showed a similar course as in the batch cultivation. However, after 216 h, a steeper decrease of the pH value occurred in the fed-batch fermentation, reaching a minimum pH of 5.74 ± 0.11 after 333 h. The final increase of the pH value in the fed-batch cultivations happened after 406 h, considerably later as in the batch process. On the whole, it can be stated that feeding with only glucose increased the long-term viability of the fungal cells but exerted no positive effect on malic acid production.

In fed-batch cultivations with additional ammonium sulfate in the feed solution (FB B/C), much higher glucose consumption and larger fungal pellets could be observed (data shown only for FB C in Figure 2). Furthermore, a considerably lower pH minimum close to 5.0 was recognized in the late phase of the cultivation. However, malic acid production did not benefit from the additional nitrogen feeds in FB B (data not shown). In fed-batch scenario C (Figures 2D–F), in which the feeds also comprised mineral salts, the highest glucose consumption and malic acid synthesis of all fed-batch cultivations occurred. However, with a maximum titer of 33.6–39.4 g/L l-malic acid, FB C did not outperform the malic acid production in batch cultivations. The highest malic acid concentrations of FB C were measured after the first or second feeding. In the further course of the fermentation, malic acid synthesis declined, although the glucose consumption was accelerated with each feeding, resulting in glucose depletion in feed phase two to five. The minimum pH of 5.19 ± 0.05 after 288 h was considerably lower as in the batch and repeated-batch cultivations. With regard to the triplicate quality, the observations made in the batch process also applied for the fed-batch cultivation: The high consistency of the glucose concentrations did not reflect in congruent malic acid titers. Additionally, the data point to a strong effect of a deviant initial glucose concentration on malic acid production (FB A3).

### Process Mode Comparison: Highest Productivity in the Repeated-Batch Process

**FIGURE 3:**
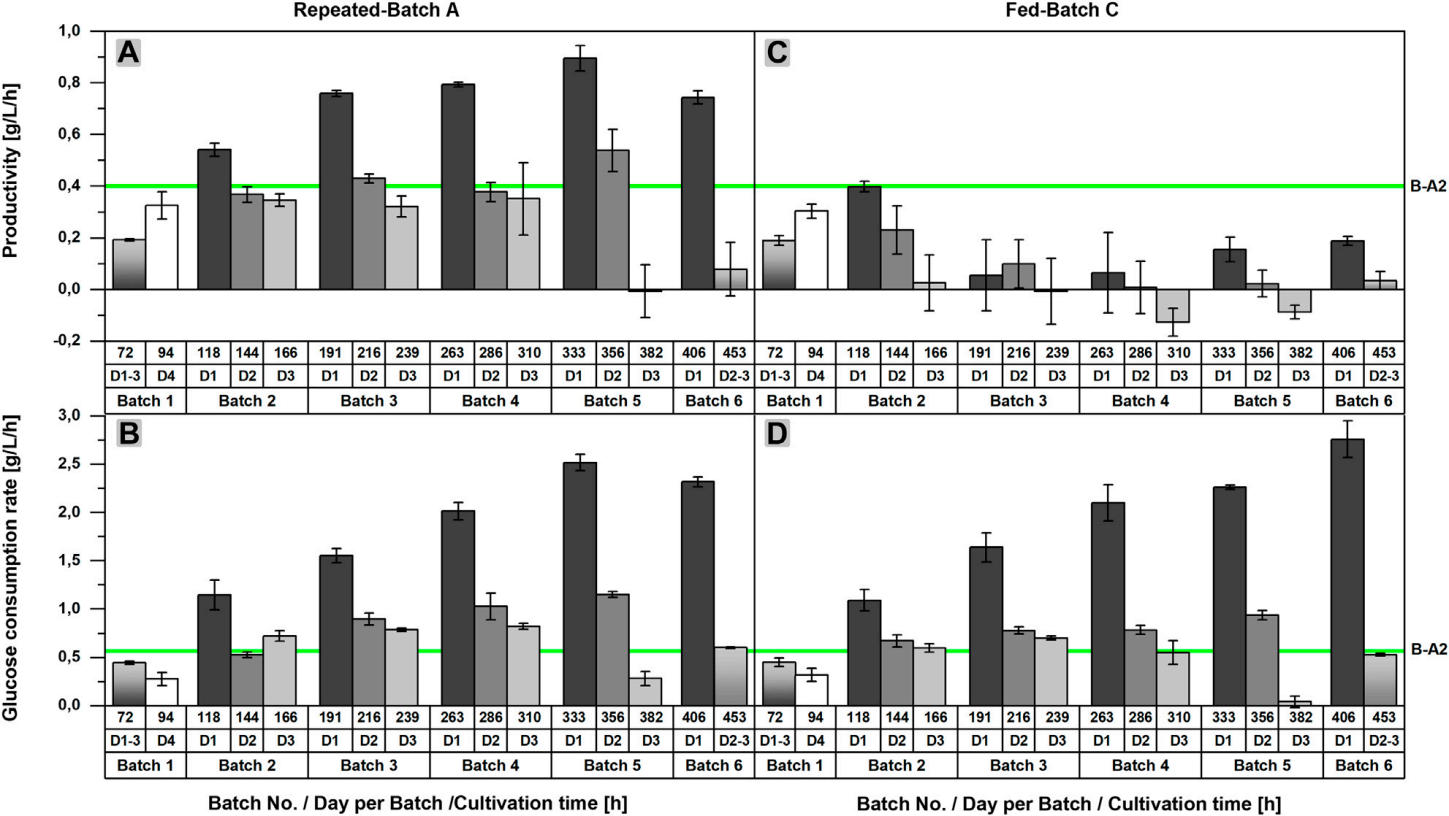
Comparison of the productivities and glucose consumption rates of batch, repeated-batch, and fed-batch cultivations for the l-malic acid production with *Aspergillus oryzae* DSM 1863. Productivities **(A,C)** and glucose consumption rates **(B,D)** between two sample points are displayed for repeated batch A **(A–B)** and fed-batch C **(C–D)**. The error bar gives the standard deviation of the mean of each triplicate. The green line indicates the maximum productivity and glucose consumption rate between two sample points determined for Batch A2.

As previously stated, an acceleration of l-malic acid production and glucose consumption after each medium exchange could be recognized in repeated-batch cultivations upon closer examination of the product and substrate concentrations. This observation becomes more obvious by looking at the productivities and glucose consumption rates of RB A, determined for each time interval between two sample points (Figures 3A,B). Regarding the productivities calculated for the first 24 h after each medium exchange, an increase from repeated-batch phase two to five was observed, reaching a maximum of 0.90 ± 0.05 g/L/h, which was more than twice as high as the maximum productivity of the batch process (0.40 g/L/h in Batch A2). Likewise, the glucose consumption rate achieved a maximum of 2.52 ± 0.09 g/L/h in the fifth phase, thereby exceeding the maximum glucose consumption rate of the batch process (0.57 g/L/h in Batch A2) by a multiple. Within each repeated-batch phase, the productivities and glucose consumption rates decreased over time. In phase five, the glucose consumption rate on day three was, especially low, as glucose became limiting. Hence, the malic acid productivity on the third day was close to zero. In the subsequent phase six, the overall productivity and glucose consumption rate decreased further.

In the fed-batch approaches with nitrogen supply (FB B,C), the glucose consumption rate showed a similar profile as observed in the repeated-batch process (Figures 3B,D). A maximum of 2.76 ± 0.19 g/L/h was achieved in the sixth fed-batch phase of FB C, which was the highest maximum glucose consumption rate of all fermentations. Differences between the initial glucose concentration of the single fed-batch and repeated-batch phases provide an explanation for the varying time points of the consumption maxima. As glucose was entirely consumed in phase five and six, the maxima were directly depending on the initial glucose concentrations and the time interval of each phase. In contrast to the glucose consumption rate, the difference between the productivites of the fed-batch and repeated-batch fermentations could not have been clearer (Figure 3A, C). The productivity of the fed-batch cultivation increased only until 118 h. With a maximum of 0.40 ± 0.02 g/L/h on day one after the first feeding, the overall productivity of the batch process was never exceeded. In the further course of the fermentation, the productivity of the fed-batch process decreased to zero and even negative productivities were calculated, presumably due to malic acid metabolization.

In sum, the parallel shake flask experiment revealed a clear advantage of the repeated-batch process over the batch cultivation with regard to the maximum productivity and a prolonged cultivation time, whereas l-malic acid synthesis could not be improved by fed-batch fermentation. The result was conclusive despite the low reproducibility of the batch process. Regarding the comparability of the fermentations, the malic acid concentrations after 72 and 94 h can give a hint on whether the starting conditions of the batch, fed-batch, and repeated-batch processes were similar. Under ideal conditions, the malic acid concentration after 94 h should be similar in all parallel fermentations, but the inoculation procedure, without biomass measurement, leaves some room for inaccuracies. The differences between the l-malic acid concentrations of the individual batch cultivations previously described already occurred after 72 h and became even more apparent after 94 h with 18.5 g/L in Batch A3 and 26.4 g/L in Batch A1. In contrast, malic acid concentrations after 94 h were much more consistent in fed-batch and repeated-batch fermentations. With an l-malic acid concentration of 20.5 ± 1.5 g/L in the fed-batch and 21.2 ± 1.2 g/L in the repeated-batch cultivation, the initial malic acid production was only slightly higher as in Batch A3 but lower as in Batch A1 and A2.

**FIGURE 4:**
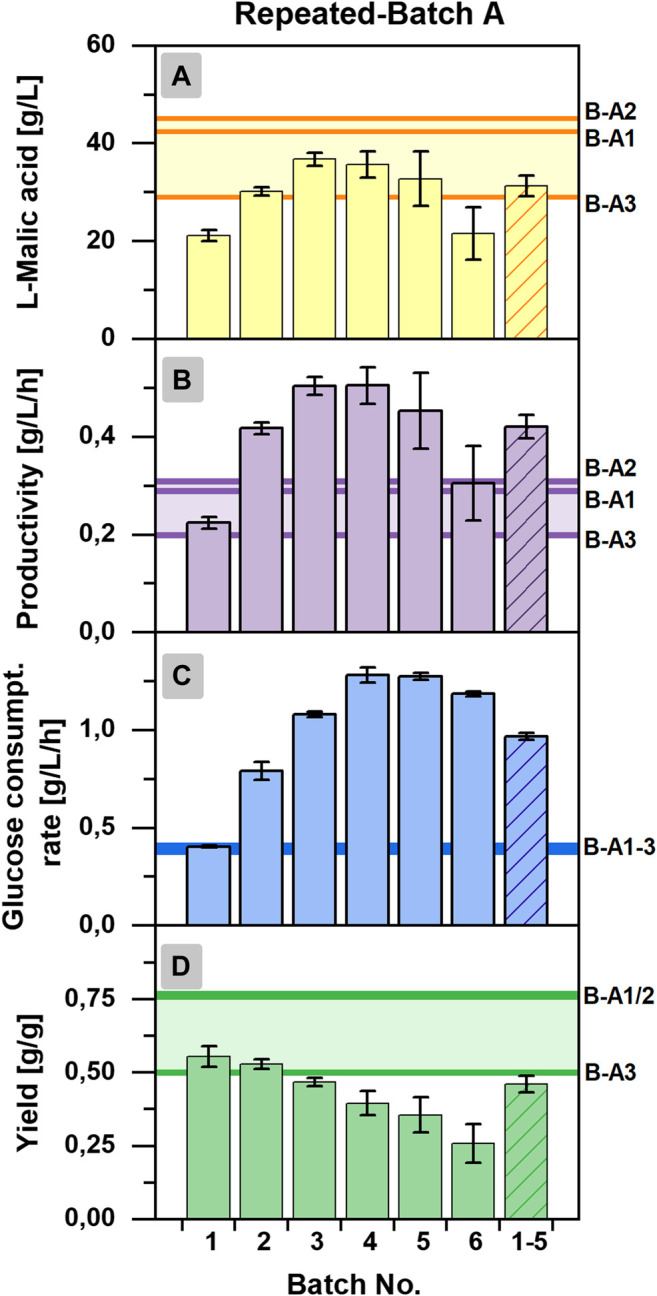
Batch-wise calculated process characteristics for the repeated-batch cultivation A with *Aspergillus oryzae* DSM 1863. **(A)** l-Malic acid concentration, **(B)** productivity, **(C)** glucose consumption rate, and **(D)** yield. The vertical lines indicate the process characteristics of each individual cultivation of Batch A (indicated as B-A1/A2/A3), calculated for a process time of 144 h.

For further analysis of the repeated-batch cultivation, performance indicators were calculated for the individual repeated-batch phase and compared to the overall titers, yields, productivities, and consumption rates determined for each individual cultivation of the batch process (Batch A1-A3) and a cultivation time of 144 h (Figures 4A–D). The l-malic acid concentration of repeated-batch phase two to five surpassed the maximum titer achieved in Batch A3 but was lower than that of Batch A1 and A2. In the context of glucose limitation, the malic acid concentration of the final repeated-batch phase fell below the titer of Batch A3. Hence, only phases one to five were considered for the mean value to obtain reasonable performance indicators for the overall assessment of the repeated-batch process. Regarding these five phases, performed within 16 days, the average l-malic acid concentration of the repeated-batch fermentation was with 31.3 ± 2.0 g/L in the lower range of the batch cultivations, whereas the average productivity of 0.42 ± 0.03 g/L/h clearly exceeded the overall productivity of all batch fermentations. The mean glucose consumption rate of the repeated-batch process was with 0.97 ± 0.02 g/L/h more than twice as high as in the batch cultivation. In the course of the repeated-batch fermentation, a steeper and longer increase of glucose consumption than the malic acid production could be observed. Accordingly, the highest yield was determined in the first repeated-batch phase and decreased over time with each new phase. With 0.46 ± 0.03 g/g, the average yield was lower as in all batch cultivations but nearly reached Batch A3.

### The Role of Nitrogen and Calcium Carbonate in Repeated-Batch Cultivations

Another experiment was performed to investigate the role of nitrogen and calcium carbonate for the l-malic acid production in repeated-batch cultivations. Due to some deviations in the experimental conditions (Table 1), RB C, D and E are not directly comparable to RB A and B. One major difference was that a faster medium exchange rate applied in RB C/D/E. The medium was replaced every 2 days, after an initial phase of 4 days, thereby the glucose concentration was kept above 0 g/L throughout the entire cultivation. RB C revealed no obvious advantage of the faster exchange rhythm and the better glucose supply on the malic acid production (data not shown), but a 3°C lower temperature in the second experiment (including RB C/D/E) might have reduced the l-malic acid production and thereby concealed a positive effect of the better glucose supply. Ochsenreither et al. reported that in bioreactor experiments, malic acid production could be considerably increased by elevating the cultivation temperature from 30 to 35°C (Ochsenreither et al., 2014). Hence, a direct comparison under identical conditions would be needed to identify the optimal exchange rhythm.

**FIGURE 5:**
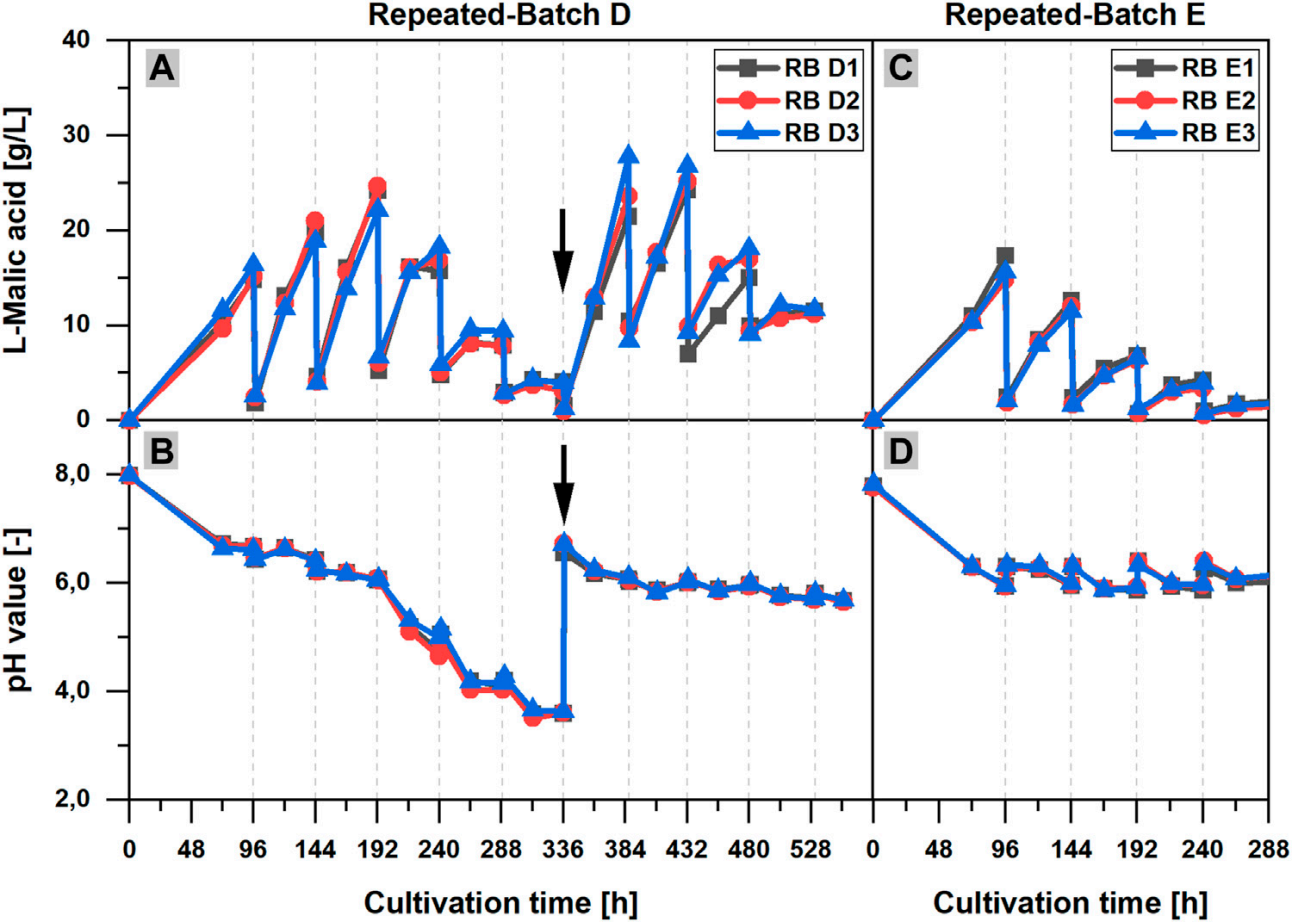
Influence of calcium carbonate and ammonium on the l-malic acid production in repeated-batch cultivations of *Aspergillus oryzae* DSM 1863. **(A,C)** l-Malic acid concentration and **(B,D)** pH value over the cultivation time for repeated-batch D **(A,B)** with ammonium in the exchange medium and calcium carbonate additions at the beginning of the cultivation and after 336 h (black arrow) and repeated-batch E **(C,D)** without ammonium in the exchange medium and regular additions of calcium carbonate. The gray vertical, dotted lines indicate the times of medium exchange for the repeated-batch cultivations.

In contrast, the important role of the calcium carbonate addition for pH stabilization and l-malic acid production could be clearly demonstrated in RB D, in which calcium carbonate was provided at the beginning of the cultivation, but was not added after the medium exchanges. Without a regular calcium carbonate supply, the pH dropped to a very low minimum of 3.59 ± 0.06 after 313 h (Figure 5B). In the first 192 h of the cultivation, the l-malic acid production increased with each repeated-batch phase, but a maximum product concentration of only 23.7 ± 1.1 g/L was already reached in phase three (Figure 5A). Afterward, the malic acid concentration decreased until 336 h and only 3.7 ± 0.4 g/L l-malic acid was measured at the end of repeated-batch phase six. Although less malic acid was produced after 192 h, the course of the pH value showed a particular steep decrease after 192 h. After the sixth medium replacement (336 h), 9 g/L CaCO_3_ was added to the shake flasks. As a consequence, the pH value was raised to 6.21 ± 0.03. Subsequently, a strong acceleration of the malic acid production could be observed, reaching 24.3 ± 2.6 g/L l-malic acid in phase seven after 387 h. In the further course of the fermentation, the pH decreased to 5.67 ± 0.01, and the malic acid production declined again with each repeated-batch phase, despite further calcium carbonate additions.

In another parallel repeated-batch fermentation (RB E), the necessity of a steady nitrogen supply for the l-malic acid production was proved. Needless to say, without ammonium sulfate in the exchange medium, the pellet growth was limited by nitrogen depletion in the initial repeated-batch phase. Moreover, the experiment clearly demonstrates that the absence of nitrogen has a significant effect on the malic acid production. The maximum product titer of 15.9 ± 1.3 g/L was already measured after 96 h, at the end of the initial repeated-batch phase (Figure 5C). Afterward, the malic acid production declined with each subsequent repeated-batch phase, whereas in the reference cultivation with full ammonium supply (RB C, data not shown) the maximum l-malic acid concentration was 21.5 g/L (RB C2) to 39.1 g/L (RB C1) after 288 h, which was significantly higher. Regarding the pH curve (Figure 5D), only a slight decrease of the pH value over time could be observed after the initial pH drop in the cultivations without ammonium in the exchange medium (RB E). The pH minimum was 6.33 ± 0.01 after 168 h, which was comparably high.

**FIGURE 6:**
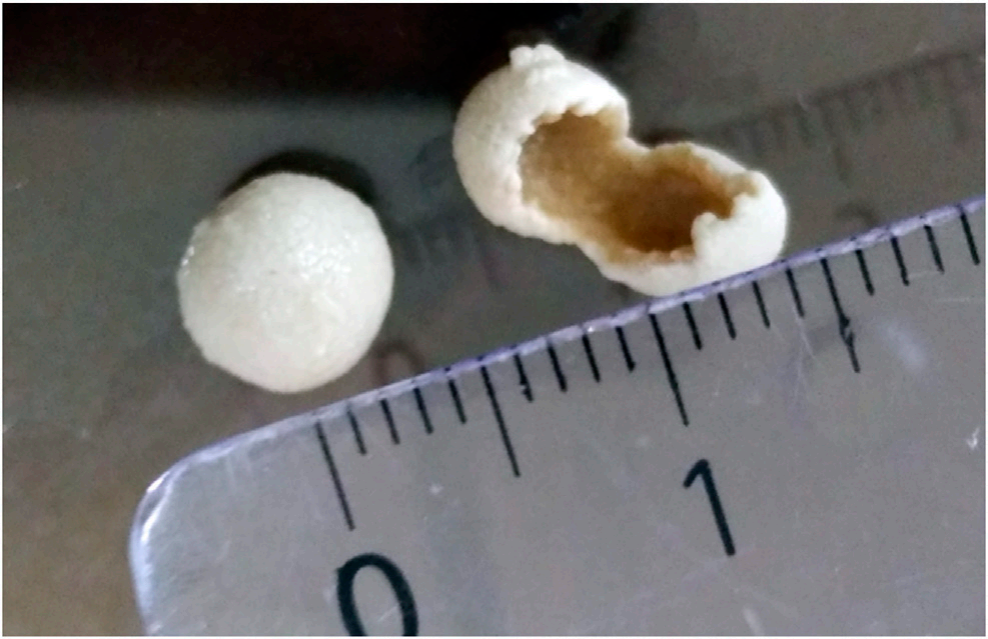
Fungal pellets of *Aspergillus oryzae* DSM 1863 with a hollow core. The picture was taken at the end of the repeated-batch cultivation RB C.

Regarding the fungal biomass, it could be observed that a steady nitrogen supply leads to a continuous pellet growth in the repeated-batch experiments (RB A/B/C), as already seen in the fed-batch cultivations (FB B/C). While the pellets reached a size of up to 8 mm, they were hollow in the inside (Figure 6), probably caused by cell degradation as a consequence of transport limitations.

## Discussion

In general, biotechnological processes can be operated as batch, fed-batch, repeated-batch, or continuously. Batch processes are the easiest to implement and therefore widely used, but in some cases alternative process modes are economically preferable. Fed-batch processes can be used to prolong the productive phase of a cultivation, especially if the carbon source cannot or should not be presented in high concentrations. Furthermore, the substrate concentration can be kept in the optimal range for product synthesis, avoiding both substrate inhibition and carbon limitation. If, on the other hand, product inhibition is present, a repeated-batch process can be advantageous, since the product concentration can be kept at a low level by replacing the medium while the biomass remains active. In this way, the production phase can be greatly extended.

In the last years, more studies dealing with l-malic acid production have been published; however, its regulation is still not well understood which renders a rationale choice of process modes and operation conditions. The present study compares the three process modes for the first time systematically in parallel shake flask cultivations and provides new insights into l-malic acid production by the natural production host *Aspergillus oryzae* DSM 1863.

### Batch Cultivation

To determine what happens during the late phase of l-malic acid production in the batch mode, the cultivation time was prolonged to ensure that the substrate will be exhausted at the end of the experiment. It has been recognized before that a high C:N ratio is mandatory for organic acid production in general and also for malic acid production in batch processes (Peleg et al., 1988; Ochsenreither et al., 2014; Ding et al., 2018), meaning that the carbon source should be provided in high excess during the process. However, to the best of our knowledge, it was not clear whether malic acid production would simply stop upon carbon source depletion, that is, malic acid concentration would stay constant, or whether it would be reassimilated, that is, malic acid concentration would decrease. In this study, malic acid was reassimilated in all batch cultivations. However, in only one of the three shake flasks, a timely correlation between glucose depletion and malic acid maximum occurred. In the other two shake flasks, the malic acid titer peaked when still more than 30 g/L of glucose was left. According to these observations, glucose depletion might be one but not the only reason for malic acid reassimilation in batch cultivations. This aspect is further illuminated by the results of the fed-batch and repeated-batch cultivations conducted in parallel, which will be discussed in the subsequent sections.

The reason for the high variances in product formation cannot be explained comprehensively by the obtained data. While being fairly comparable after the first 72 h, differences became more prominent thereafter. Given the similar consumption of glucose, the question arises, if other products are formed, which could explain the findings. Biomass formation as carbon sink is unlikely since same amounts of nitrogen were provided for each culture and were regularly depleted after about 48 h. Hence, other explanations are needed. *A. oryzae* has a complex morphology, which is not well understood (Papagianni, 2004). Under our culture conditions, *A. oryzae* grows in the form of large pellets with a size of several millimeters. Accordingly, mass transfer limitations, cell differentiation, or aging processes, and therefore changes in cell metabolism are likely. For l-malic acid production with *A. oryzae*, Chen et al. proved the general importance of cell morphology (Chen et al., 2019).

In terms of pH, batch culture A3 is particularly interesting. The pH value declined further after malic acid reassimilation started, thereby differing from the other two cultures. Since we applied the same amount of calcium carbonate in all cultures and did not observe any differences in glucose consumption, lower malic acid production, and different pH minima might also indicate changes in by-product formation. This aspect should be investigated in more detail for *A. oryzae* in subsequent studies.

### Fed-Batch Cultivations

Fed-batch scenario A proved that glucose limitation is not the decisive limiting factor in l-malic acid production with the *A. oryzae* wild-type strain. Although glucose was fed regularly, keeping the substrate concentration above 40 g/L at all times, malic acid concentration was consistently lower as the maximum titer achieved in the batch cultivations. While malic acid was continuously synthesized after each feeding, the low level of production could not compensate for the dilution effect of the feeding, resulting in decreasing malic acid concentrations in the course of fermentation. However, it is noteworthy that the viability of the cells was prolonged in the fed-batch mode. The characteristic pH increase indicated cell degradation only after 17 days (408 h).

In prolonged fed-batch fermentations, the viability and productivity of the cells might profit from additional nutrient supply. This was investigated in fed-batch scenario B, in which carbon and nitrogen was fed, and scenario C, in which the complete medium was supplemented. In both cases, accelerated glucose consumption and lower pH values were observed, both probably due to biomass growth after nitrogen uptake (data of FB C in Figure 2). Biomass was not determined; however, pellet growth was noticed. A prolonged viability could not be recognized; the pH rise occurred in FB C after 17 days and was comparable to FB A. The much higher overall glucose consumption did not reflect in a comparable increase of the malic acid production in FB B and C. In this case, biomass as carbon sink is likely, as continuous pellet growth could be observed. The slight increase of the overall product titer was, therefore, achieved on the cost of a strong reduction of the yield (Table 2). Hence, the fed-batch process seems not to be beneficial for l-malic acid production with the wild-type *A. oryzae* strain.

Our results seem to be in conflict with the study of Liu et al. (2017), in which only glucose was supplied during fed-batch and much higher malic acid concentrations were reached than those in the batch fermentation. However, Liu et al. (2017) used a genetically modified strain instead of the wild-type. By overexpression of C4-carboxylic acid transporters, Liu et al. (2017) achieved a significant increase of the malic acid titers compared to the wild-type, confirming the results of a previous study by Brown et al. (2013). Accordingly, product inhibition could be a decisive limiting factor for malic acid production in *A. oryzae* wild-type strains. This might explain why the maximum malic acid concentration of the batch process in our study with the wild-type strain could not be improved by fed-batch cultivation, regardless which feeding scenario was applied.

Product inhibition depends on not only the acid concentration but also the ratio of the undissociated form, which could re-enter the cell *via* diffusion and disturb pH homeostasis and cause a loss of energy (Zambanini et al., 2016). For malic acid with pK_a_ values of 3.40 and 5.20, the ratio of the undissociated form increases below pH 7. Therefore, it is noteworthy that another difference to our study was the constant pH value of 6.0 as in the work of Liu et al. (2017), since both batch and fed-batch cultivations were conducted in a bioreactor with pH regulation with sodium carbonate in addition to the buffering with 90 g/L CaCO_3_. However, in calcium carbonate buffered FB A (glucose only feed), the pH decreased only slightly below six in the later feeding phases. In contrast, in FB B and FB C, a stronger decrease of the ambient pH down to a minimum of 5.0–5.2 could be observed in consequence of the steady nitrogen consumption. The low pH values and glucose limitation in the later fed-batch phases could have impeded malic acid production. Hence, additional fed-batch experiments with better pH control and sufficient glucose supply would be interesting to complete our findings. Further studies should also include quantification of possible acidic by-products and nitrogen consumption, as deviations between the pH curves of the individual fed-batch cultivations were observed that did not reflect in increased malic acid synthesis.

In general, the complex interplay between organic acid accumulation in the cell, internal and external pH values, transport processes, and the regulation of the metabolism in filamentous fungi are not sufficiently understood (Vrabl et al., 2012). However, avoiding (malic) acid accumulation in the cytosol and the control of the ambient pH are known key factors for prevention of product inhibition. Genetic manipulation (Liu et al., 2017) is not the only option to improve malic acid production by *A. oryzae*. Lowering the malic acid concentration in the medium can also prevent product inhibition, as a concentration gradient could facilitate the malic acid export. Although the role of calcium carbonate in organic acid production is not completely clear, product removal by precipitation of the acids as calcium salts is supposed to contribute to improved organic acid production with increased calcium carbonate concentrations, ensuring a pH above 6 (Geyer et al., 2018). In our study, we investigated the impact of calcium carbonate only in repeated-batch cultivation, which is discussed in the following section. The repeated-batch process itself can also reduce product inhibition by lowering the malic acid concentration in the medium. Our results for the repeated-batch cultivation of *A. oryzae* are discussed in the next section.

### Repeated-Batch Cultivations

In the literature, the advantages of the repeated-batch process for microbial organic acid production were already described for different acids and production strains. For example, Park et al. reported an extended itaconic acid production in shake flask cultivations with *A. terreus* for 45 days in nine repeated-batch cycles. The productivity (0.47 g/L/h) was 1.5 times increased compared to the 7-day batch process (Park et al., 1994). In another study, *A. niger* cells immobilized in Ca-alginate gel beads retained the ability to produce citric acid for up to 84 days in repeated-batch shake flask fermentations (Roukas, 1991). However, studies on the repeated-batch cultivation for l-malic acid production are missing.

This study closes the literature gap with results from several repeated-batch cultivations of *A. oryzae*, carried out in shake flasks under different conditions with cell recycling by decantation. The results for l-malic acid production are comparable to the observations of Park et al. regarding itaconic acid. The average productivity of the repeated-batch cultivation (0.42 ± 0.03 g/L/h) with a runtime of 16 days (5 cycles) was 1.5-fold higher than that of the batch process (6 days), while malic acid concentration and yield was more or less similar. The repeated-batch process was stopped after six cycles and 19 days, due to decreasing productivities, most likely caused by a temporary glucose limitation in later repeated-batch phases. However, malic acid production could be restored with the next medium exchange, proving the robustness of the process. As longer runtimes at high productivities could reduce operation costs, it would be interesting to see if a sufficient glucose supply would enable a further prolongation of the malic acid production or if other factors would become limiting in extended repeated-batch experiments. Additionally, the usage of immobilized cells in repeated-batch cultivations could be an interesting topic for further studies, as there are promising results for kojic acid production with immobilized *A. oryzae* (Kwak and Rhee, 1992).

Although the repeated-batch cultivation proved to be advantageous for l-malic acid production of the wild-type strain compared to the batch and fed-batch processes, higher productivities, titers, and yields were reported for batch and fed-batch cultivations with metabolic engineered or mutant strains of *A. oryzae*. So far, the highest productivity was reported by Liu et al. (2017) in 3 L fed-batch cultivation (1.38 g/L/h) with a multiple engineered strain. However, a direct comparison with the results of Liu et al. could be misleading due to the usage of peptone and the fact that complex nitrogen sources are known to increase malic acid production significantly compared to cultivations on ammonium sulfate (Knuf et al., 2013; Ding et al., 2018). This also applies for most of the other studies regarding malic acid production with *A. oryzae*. Only in the studies of Knuf et al. and Ding et al., ammonium sulfate was used as a nitrogen source (Knuf et al., 2014; Ding et al., 2018). Knuf et al. achieved a productivity of 0.34 ± 0.06 g/L/h with the wild-type strain *A. oryzae* NRRL 3488 in 2.7-L bioreactor batch cultivations and a productivity of 1.05 ± 0.13 g/L/h under the same conditions with the engineered strain *A. oryzae* NRRL 3488 2103a-68. Ding et al. (2018) reported a productivity of 0.57 g/L/h in 7.5 L fed-batch cultivation with a mutant *A. oryzae* strain. The studies of Liu et al. and Knuf et al., demonstrate the power of metabolic engineering and the impact of product inhibition as in both studies the overexpression of transporter genes resulted in a strong improvement of the malic acid synthesis. A closer look at our results reveals that the repeated-batch process might not reduce the product inhibition sufficiently, as the productivity was the highest directly after each medium exchange with an overall maximum of 0.97 ± 0.02 g/L/h but decreased significantly with increasing product concentration in the course of each repeated-batch phase (Figure 3). However, the maximum productivity clearly shows the potential of the wild-type strain. In further studies with the wild-type strain, the inhibitory concentration of malic acid under typical cultivation conditions should be determined.

The observed decreasing productivities within each repeated-batch phase were accompanied by a slight decrease of the pH value, as a result from nitrogen consumption and acid production, but the pH value was above six at all times due to regular calcium carbonate additions with each medium exchange. The buffering agent stabilizes the pH value and should reduce product inhibition by keeping the malic acid concentration in the media low *via* precipitation of the acid as calcium salts. Moreover, the additional calcium and CO_2_ supply *via* calcium carbonate might also have a positive effect on the malic acid production. In a study that investigated the role of calcium carbonate in the l-malic acid production with *A. oryzae* from acetate, the authors concluded that the formation of Ca-malate might have been the main function of calcium carbonate (Kövilein et al., 2021), whereas its role as a CO_2_ donor and buffering agent was assumed to be less important. However, for malic acid production on glucose, all functions of calcium carbonate could be beneficial. While the individual impact of the different calcium carbonate functions on the malic acid synthesis is still not sufficiently understood and should be investigated further, the positive impact of calcium carbonate on l-malic acid production is undoubted. Geyer et al. (2018) previously reported a correlation between malic acid production and the calcium carbonate amount, and the comparison of the overall malic acid production in repeated-batch scenario A and B (Table 2) seems to confirm this. Furthermore, repeated-batch scenario D clearly proved the necessity of regular calcium carbonate additions during repeated-batch cultivations. Without regular calcium carbonate supply, the productivity and pH value decreased with each phase, but malic acid synthesis could be fully restored by adding calcium carbonate in a later stage of the cultivation, while the pH was increased to more than six. All in all, repeated-batch scenario D demonstrates the pH tolerance of *A. oryzae*, while the productivity clearly depends on calcium carbonate. However, the usage of calcium carbonate has several drawbacks, such as an increased viscosity of the cultivation broth and issues in downstream processing (Li and Xing, 2017; Koevilein et al., 2020). Therefore, *in situ* product recovery combined with a regular nutrient feeding would be interesting for prolonged malic acid production with the wild-type strain. Processes with *in situ* product recovery already proved to be beneficial for itaconic acid (Kreyenschulte et al., 2018) and fumaric acid production (Cao et al., 1996; Xu et al., 2017). Malic acid can be separated by reactive extraction with, for example, tri-octylamine (Uslu and Kirbaslar, 2009; Uslu and Kırbaşlar, 2010), which was already successfully tested for *in situ* recovery of itaconic acid from a fermentation broth with *A. terreus* (Kreyenschulte et al., 2018). Accordingly, it would be interesting to test this option also for the malic acid production with an *A. oryzae* wild-type strain. Separation *via* adsorption columns or electrodialysis could be further options for *in situ* product recovery of malic acid (Lameloise and Lewandowski, 2012; Liu et al., 2014; López-Garzón and Straathof, 2014; Pandurić et al., 2017). To get a clearer picture of the optimal pH value for the malic acid production and its impact on the spectrum of acidic by-products, bioreactor cultivations with pH statization by a liquid titration agent should be evaluated in a subsequent study.

Besides the influence of calcium carbonate and pH value, the nitrogen–carbon ratio is known to have a strong impact on growth and l-malic acid production in batch and fed-batch cultivations (Ochsenreither et al., 2014; Liu et al., 2017; Ding et al., 2018; Ji et al., 2021). In our study, repeated-batch scenario E proves that a steady supply of nitrogen in the production phase is necessary for a prolonged malic acid synthesis with high productivities. The improved nutrient supply in the repeated-batch cultivations (A–D) in combination with reduced product inhibition resulted in higher productivities, than the (fed-) batch cultivations. The addition of nitrogen and other nutrients enables biomass formation, keeping the outer cells of fungal pellets juvenile and might therefore prevent metabolic differences due to differences in aging processes. Although the relationship between productivity and fungal morphology is not entirely understood (Papagianni, 2004; Krull et al., 2013), small sized pellets are supposed to be optimal for malic acid production, due to a limited mass transfer into the core of larger pellets (Chen et al., 2019). For larger pellets with a hollow core (Figure 6), our study indicates that an increase of pellet size obtained by a steady nitrogen supply reflects in higher productivities. This might be caused by the larger pellet surface with an increased number of active cells that do not face any transport limitation.

A drawback of the steady pellet growth is the corresponding reduction of the yields over time, as more and more glucose was used for biomass formation. The average yield for five repeated-batch phases (0.46 ± 0.03 g/g) was already lower as in the batch process and would decrease further in prolonged cultivations. Hence, a further increase of cultivation runtime and productivity would be on the expense of lower yields. To compensate the reduction, the formation of by-products needs to be determined and reduced. Key parameters that impact the side-product formation are the pH value (Vrabl et al., 2012) and the C/N ratio (Ochsenreither et al., 2014; Ding et al., 2018; Ji et al., 2021). While Ochsenreither et al. tested the C/N ratio for the batch cultivation of the wild-type strain, which was also used in this study, Ding et al. and Ji et al. evaluated the C/N ratio and nitrogen supply strategy for fed-batch cultivations of different mutant strains. These literature insights are not directly transferable to the repeated-batch process with the wild-type strain but should be considered for the optimization of the nitrogen and glucose supply of the repeated-batch cultivation. For a deeper understanding and further optimization of the repeated-batch process, a comprehensive analysis would be required, including by-product detection and biomass measurement. Furthermore, the ratio between living and dead biomass could be an important parameter, worth investigation.

In conclusion, our systematic comparison of different process modes for the l-malic acid production with the wild-type strain *A. oryzae* DSM 1863 showed that the repeated-batch process was clearly advantageous compared to batch and fed-batch processes with regard to the productivity and a prolonged cultivation time. Furthermore, the study demonstrates that sufficient glucose, nitrogen, and calcium carbonate supply during the repeated-batch process is required to achieve high productivities in prolonged cultivations. In a next step, pH control, calcium carbonate supply, and the C/N ratio should be optimized. To achieve higher yields, by-products should be reduced, and the biomass-related productivity should be increased.

To our knowledge, this study is the first evaluation of a repeated-batch process for the l-malic acid production with *A. oryzae.* Next, repeated-batch cultivations could be tested for the malic acid production with genetically modified high-performance strains to enable prolonged cultivations with high productivities, titers, and yields. Due to the drawbacks of the calcium carbonate for downstream processing, *in situ* product recovery, for example, *via* reactive extraction or electrodialysis, should also be considered an alternative process strategy to overcome product inhibition.

All in all, the study underlines the potential of *A. oryzae* for the l-malic acid production and gives valuable insights and new starting points for further process development toward an economic viable fungal malic acid production process.

## Data Availability

The raw data supporting the conclusion of this article will be made available by the authors, without undue reservation.
